# Urbanization drives convergence in soil profile texture and carbon content

**DOI:** 10.1088/1748-9326/abbb00

**Published:** 2020-10-14

**Authors:** Dustin L Herrmann, Laura A Schifman, William D Shuster

**Affiliations:** 1 Oak Ridge Institute for Science and Education Research Participant Program with National Risk Management Research Laboratory, U.S. Environmental Protection Agency, 26 W. Martin Luther King Dr., Cincinnati, Ohio 45268, United States of America; 2 National Research Council Research Associate Program with National Risk Management Research Laboratory, U.S. Environmental Protection Agency, 26 W. Martin Luther King Dr., Cincinnati, Ohio 45268, United States of America; 3 National Risk Management Research Laboratory, U.S. Environmental Protection Agency, 26 W. Martin Luther King Dr., Cincinnati, Ohio 45268, United States of America; 4 Current affiliation: Department of Botany and Plant Sciences, University of California, Riverside, CA 92 521, United States of America; 5 Current affiliation: Massachusetts Department of Environmental Protection, 1 Winter Street, Boston MA 02108, United States of America; 6 Current affiliation: College of Engineering, Wayne State University, 5050 Anthony Wayne Drive, Detroit, MI 48 202, United States of America

**Keywords:** urban ecosystem, urban critical zone, soil carbon, soil particle size, convergence

## Abstract

Urban development has driven extensive modification of the global landscape. This shift in land use and land cover alters ecological functioning, and thereby affects sustainable management agendas. Urbanization fundamentally reshapes the soils that underlay landscapes, and throughout the soil profile, extends impacts of urbanization far below the landscape surface. The impacts of urbanization on deeper soils that are beyond the reach of regular land management are largely unknown, and validation of general theories of convergent ecosystem properties are thwarted by a dearth of both level of measurement effort and the substantial heterogeneity in soils and urban landscapes. Here, we examined two soil properties with strong links to ecological functioning—carbon and mineral-fraction particle size—measured in urban soils, and compared them to their pre-urbanization conditions across a continental gradient encompassing global soil diversity. We hypothesized that urbanization drove convergence of soils properties from heterogeneous pre-urban conditions towards homogeneous urban conditions. Based on our observations, we confirm the hypothesis. Both soil carbon and particle size converged toward an intermediate value in the full data distribution, from pre-urban to urban conditions. These outcomes in urban soils were observed to uniformly be fine textured soils with overall lower carbon content. Although these properties are desirable for supporting urban infrastructure (e.g. buildings, pipes), they constrain the potential to render ecosystem services. Since soil profile texture and carbon content were convergent and observed across 11 cities, we suggest that these property profiles can be used as a universal urban soil profile to: 1) provide a clear prediction for how urbanization will shift soil properties from pre-urban conditions, 2) facilitate the adoption of commonly-accepted soil profiles for process models, and 3) offer a reference point to test against urban management strategies and how they impact soil resources.

## 1. Introduction

The previous and present century are hallmarked by human population growth and rural-to-urban migration with urbanization currently being the most rapidly expanding land use globally (Seto et al 2012, Mertes et al 2015). Conversion to urban land uses alters the pre-existing landscape substantially as it typically involves extensive modification to soils through grading, excavation, and filling activities (Jones et al 2014, Herrmann et al 2018). Urbanization transforms both natural and social histories of soils. The urbanization process marks an indelible transformation of soils, imprinting development onto soils and their underlying landscapes. The transformations involved in urban development take place over short time-frames, which is contrasted with that of soil formation, which takes place over millennia (Lo Papa et al 2011, Herrmann et al 2018). Along with structural changes due to urbanization, the ecological functioning of soil profiles is also expected to change. Understanding how shifts in structure lead to altered functioning is key to planning for desirable sustainability, resilience, and equity outcomes (Childers et al 2015, Herrmann et al 2016). Since soils form the basis of terrestrial ecosystems, there is great urgency in understanding how soils are being modified by urbanization and thereby better inform ecological management schemes.

Urban soils have become a significant focus of research activity (Effland and Pouyat 1997, Scharenbroch et al 2005, Pavao-Zuckerman 2008, Pouyat et al 2010, Raciti et al 2011, Trammell et al 2011, Edmondson et al 2012, Herrmann et al 2017). However, there are large gaps in our knowledge of urban soils. This is attributed to a historic preoccupation with agricultural soils, such that major 20th century soil surveying efforts excluded urbanized landscapes. The present urban soils research is often limited to surface soils, tied to specific land uses (e.g. residential lawns in one neighborhood or city), or lacking in suitable soil profile reference conditions (i.e. pre-urban) for identifying and partitioning change due to urbanization. Having addressed these limitations, Herrmann et al (2018) revealed the widespread loss of soil horizons at intermediate depths (30–80 cm) across a continental gradient of cities and land uses. The comparative approach taken therein offers promise for uncovering the major patterns in how urbanization has shaped soil profiles that go beyond classifications offered by land uses. In this paper we move beyond the horizon level and uncover soil profile patterning in soil texture and carbon content.

Urbanization acts on soils that formed under natural conditions as native soils, or those soils with a prehistory of agricultural management. Pre-urban profiles are likely to approximate theoretical vertical distributions of soil properties in a given profile. In soil development, components of soil start as an isotropic (e.g. unweathered rock or till) vertically-uniform distribution, and through time, soil formation and disturbance rearranges soils to an anisotropic profile as soil properties develop more variation in vertical directionality (Jobbágy and Jackson 2000, 2001). For example, soils develop intermediate layers that are relatively high in clay content as finer soil material translocates from surface soils to form an accumulative intermediate layer. The theoretical profile for soil particle size presents with finer texture particles at intermediate depths and coarser, unweathered particles near the surface and deep in the profile (Minasny et al 2016). The typical soil profile for soil carbon content is high at the surface where biological activity is greatest, and theoretically, declines exponentially with depth (Minasny et al 2016).

Shifts in soil properties along the soil profile are difficult to attribute to any one impact, as profiles exhibit extensive heterogeneity that forms through time. Therefore, it is key to identify reference profiles as a basis for comparison. Ideally, soil profiles would be observed both before and after urbanization activities occur. Alternatively, pre-urbanization reference profiles can be approximated by using expert knowledge and archival soil surveys to re-construct the pre-urban soil series, which is analogous to the species level of taxonomy in biology. Major differences arise at the regional scale, drawing from the soil forming factors of time, climate, geological formation or parent material, topography, and biological activity (Bockheim 2005). Significant variation within a region, local landforms (Milne 1936, Huggett 1975), and geologic histories (Bockheim 2005) suggest sampling along a continental gradient. To account for this, paired pre-urban reference and urbanized soil profiles are then sampled across soil orders.

Previous studies on urban soil biophysical properties, and especially urban soil carbon, have shown that urbanization creates novel patterns in soil profiles (Schifman et al 2018, Trammell et al 2019, Canedoli et al 2019). Based on previous work, there is a consistent literature indicating widespread urban ecological homogenization, where ecosystem-level properties (e.g. water fluxes, presence-absence of species across cities) (Mckinney 2006, Groffman et al 2014, Pouyat et al 2015, Hall et al 2016, Schmidt et al 2017) converge to common levels within and among cities. Strongly influenced by management, there is evidence that surface soil carbon content in lawns converges to an intermediate level across cities (Pouyat et al 2009, Smith et al 2018, Trammell et al 2020). In soils, we posit that convergence can be observed in at least two different ways: the quantities and distributional characteristics of soil properties, and the degree of anisotropy in soil properties along the soil profile.

An outstanding question is whether urbanization acts to generally homogenize soil properties to drive convergence in soil profiles within and among cities. Here, we present a continental-scale examination of urbanization effects on the distribution of two soil properties—texture as mineral-fraction soil particle size and carbon content—along the vertical soil profile to a depth of 1.5 m. Texture and carbon are the key physical and biological indicators of soils, respectively, and as such are most indicative of likely impacts on ecosystem functioning and ecological management outcomes of shifts with urbanization processes.

## 2. Methods

### 2.1. Urban and reference soil database

We field sampled urban soils collecting 332 soil cores in 11 cities from 2010–2016 (Herrmann et al 2018), including: Atlanta, GA (ultisols); Camden, NJ (aquic spodosols); Cincinnati, OH (unglaciated alfisols); Cleveland, OH (aquic alfisols); Detroit, MI (alfisols, mollisols); New Orleans, LA (vertisols, histosols); Omaha, NE (ustic alfisols and mollisols); Phoenix, AZ (aridisols); Portland, ME (upland spodosols); Tacoma, WA (andisols); and San Juan, PR (oxisols). The urban soils assessment sampled soils from a range of land uses within the urbanized landscape in each city. Land uses included: residential properties, parks, schools, vacant lots, and public rights-of-way. As our objective was a comprehensive survey of urban soils, our sampling strategy was to capture the many land uses that are considered urban and not a comparison among them, although they can influence soil properties (Schleuß et al 1998). Preliminary analyses also indicated land use was not an important driver of soil characteristics in our dataset.

We built a correspondent database of reference soils using Official Series Descriptions from the USDA-NRCS National Cooperative Soil Survey database. The reference soil series selected to pair with an urban profile was chosen based on the likely soil series to exist in the location of the urban sample prior to urbanization. The likely pre-urban reference soil series was determined from soil maps and based on the expertise of USDA soil scientists familiar with soils of the region. Data on soil properties was collected at the horizon level and, for our analyses, resolved to 1 cm depth slices. Although urban soil cores were observed to an average of 4 m depth, due to standard soil survey procedures, the pre-urban reference data set— and thus the comparison—was limited to 150 cm depth. Both datasets are available for download through the United States government data repository at https://catalog.data.gov/harvest/about/epa-sciencehub. All data organization, analysis, modeling, and visualization was completed in base R (R Core Team 2016) and R packages *aqp* (Beaudette et al 2013) for wrangling and preliminary visualization of horizon-structured soils data, *dplyr* (Wickham and Francois 2016) and *tidyr* (Wickham 2017) for general data wrangling, and *ggplot2* (Wickham 2009) for producing graphics.

### 2.2. Soil property quantification

We determined two soil properties, soil texture (as mineral-fraction geometric mean diameter), and carbon content. Soil textural class (e.g. silt loam) was determined in the field for urban soils by a consulting soil scientist (S. Dadio, Cedarville Engineering Group, LLC, North Coventry, Pennsylvania, USA) using the texture-by-feel method (Thien 1979). Soil textural class was converted to centroid percentage for each of the soil textural separates (sand, silt, and clay) as per the USDA soil texture triangle (supplementary materials available online at stacks.iop.org/ERL/15/114001/mmedia). The three-dimensional sand, silt, and clay representation of particle size was transformed into a one-dimensional quantification of particle size as the geometric mean particle diameter, hereafter *particle size*, following Shirazi and Boersma (1984).

We predicted carbon content from Munsell soil color, specifically value, which we had for all horizons from the total carbon content analysis (combustion technique on an elemental analyzer, EA1110 CHNSO Analyzer, CE Instruments, Wigan, UK) we had complete on a subset of horizons (suppl. Materials). Munsell soil color value was correlated with total soil carbon as:

$$\mathrm{Log}\;\left(\mathrm{Total Carbon}\right)=5.07-0.58^{*}\mathrm{Munsell Value}$$

We used this relationship (p < 0.01, *r*^2^ = 0.23, *n* = 671) to predict total carbon content (mass basis) for all horizons in the urban and reference dataset. We quantified uncertainty in modeled carbon concentrations by performing a similar analysis of prediction on a particularly dense data set (Detroit MI), for which carbon content was measured for all urban (*n* = 57 soil cores, 369 soil layers) and reference (*n* = 21 pedons, 154 soil layers) sites, and within each site, all soil horizons to 150 cm below ground surface (suppl. Materials).

### 2.3. Profile anisotropy

The vertical distribution of soil properties is an indicator of soil development, and represented by the unitless Index of Profile Anisotropy (IPA) metric developed by Walker and Green (1976) using the equation:

(4) $$IPA_{i}=\frac{\sum \left|\sigma \right|}{\mu }$$

where σ is the variance of a given soil property at each 1-cm slice of the soil profile, and μ is the overall mean of the soil property for the given core, calculated over the entire 150 cm depth. IPA values of 0 indicated entirely uniform properties along the vertical soil profiles. Because the particle size and carbon content values were derived from texture classes and Munsell values that contain a range of levels, we considered that profiles with soil property sample variance of zero arose out of limits on analytical methods, rather than the profiles themselves interpreted as entirely homogenous. In these cases, zeroes were replaced with a minimum IPA value so as to avoid singularity in the ratio urban IPA:reference IPA. The minimum IPA was designated as the lower quartile (25%) of the median reference IPA value for each soil property, which is 13.6 for particle size and 10.7 for carbon content.

### 2.4. Quantifying convergence

Convergence occurs when urbanization increases lower preurban values and decreases higher preurban values towards a common intermediate value. To quantify this, we regressed the ratio of urban-to-reference values for each variable (i.e. particle size, carbon, and profile IPAs) against the reference values. A ratio greater than 1 means the urban value was greater than its preurban reference value and a ratio of less than 1 means the urban value was less than its reference. We define the point of convergence as the reference value at which the urban-to-reference ratio equals 1 according to linear regression. Patterns of convergence in soil properties were quantified with linear models for each 1 cm depth slice from 0–150 cm soil depth, testing the relationship between log-transformed, pre-urban property levels (dependent) to the log-transformed ratio of urban to pre-urban soil property values (independent variable). Modeled converged urban soil profiles were constructed for particle size and carbon levels from back-transformed extracted values from each 1 cm depth slice linear regression for the pre-urban level at which urban to pre-urban ratio equaled 1, i.e. the point of convergence. Convergence in profile anisotropy was tested by the same setup with the exception that there is only one IPA value for each soil profile and not one for each depth slice.

## 3. Results

**Figure1.**
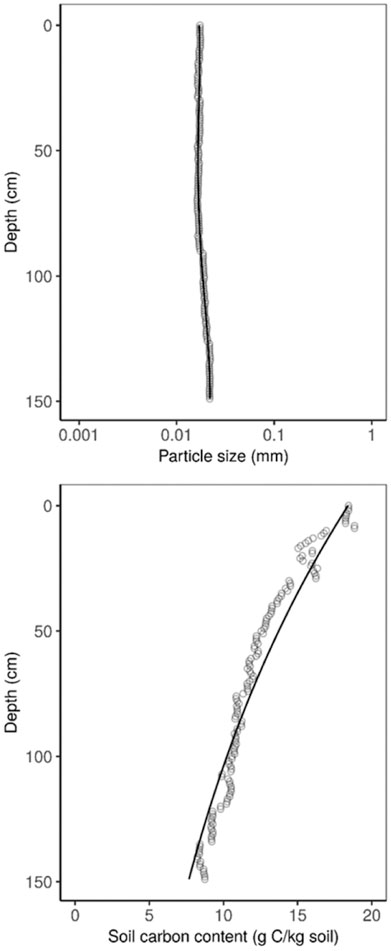
Urban soil property profiles for particle size (mm, log scale; geometric mean diameter of soil mineral fraction) and soil carbon content. Circles are the converged soil property value (i.e. intemediate value towards which urban soils were approaching relative to pre-urban reference soil) at each 1–cm depth slice from 0–150 cm soil depth. Lines are derived from regression models. Particle size model was derived as a standard fourth-order Polnomial (*y* = − 4.01 + 0.10x + 0.59x^2−^0.06x^3−^0.18x^4^; adj. *R*^2^ = 0.95; *F*_df=145_ = 733) An exponential function was applied to drive the carbon profile [*y* = C_o_ *exp(−0.005868*x; p < 0.001, residual SE = 0.8, df = 149; where C_o_ is the mean carbon content at the soil surface, equal to 18.4 g C kg^−1^ soil in this model].

Based on linear models for each 1 cm depth slice, we found urbanized particle size and total soil carbon either increased or decreased from pre-urban levels resulting in both soil properties being converged toward intermediate values relative to their data distributions (for all 1 cm depth slices and both soil properties, p < 0.01). The tendency for urbanization to drive convergence in particle size and soil carbon content is visualized in figure 1. Urbanized particle size profiles had a uniform vertical distribution across the entire profile (figure 1). The particle size profile (figure 1) was modeled as a standard fourth-order polynomial (*y* = − 4.01 + 0.10x + 0.59x^2−^0.06x^3−^0.18x^4^; adj. *R*^2^ = 0.95; *F*_df=145_ = 733). This is the simplest model to form a theoretical, reference particle size profile (Minasny et al 2016). This pattern was most evident for clayey to silty particle sizes. We did not confirm predicted translocation of finer materials to intermediate soil horizons, however, this is consistent with results from Herrmann et al (2018), which showed that urbanization prevented the formation of intermediate soil horizons, a feature of which is enrichment with fine soil material via illuviation processes. The vertical distribution of soil carbon content approximated an exponential decline (figure 1). Following the carbon profile model of Russell and Moore (1968), we applied an exponential function to derive the carbon profile (figure 1; *y* = C_o_*exp(−0.005868*x); p < 0.001, residual SE = 0.8, df = 149; where *C*_o_ is the mean carbon content at the soil surface, equal to 18.4 g C·kg^−1^ soil in this model).

**Figure 2.**
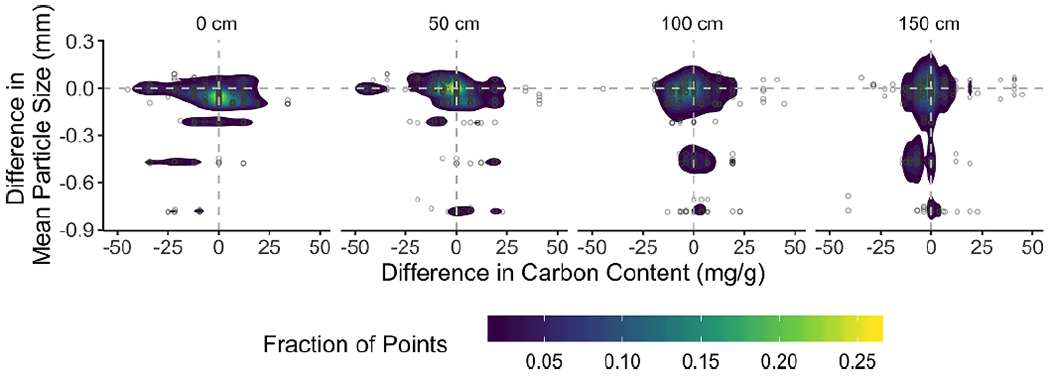
Differences between urban and rural soil in carbon and mean particle size at four depths slices that highlight where the qualitatively important shifts in soil properties take place. Carbon shifts are most pronounced at the surface and up to 50 cm depth, where particle size differences are substantial throughout the profile. Plots are colored based on the density of points represented.

Urbanization drove changes in the vertical distribution of particle size and soil carbon through integration of: i) the direction or trajectory of shifts in each soil property throughout the profile, and ii) change in profile anisotropy. First, we examined shifts in particle size and carbon content across the entire 0–150 cm profile and present visualizations for 0, 50, 100, and 150 cm profile depths (figure 2) which represent the major changes across the entire profile. Qualitatively, urbanization drove soil profiles toward smaller particle size in urban compared to pre-urban soils by up to 0.8 mm at all depths and overall decreased carbon content (figure 2). However, shifts in particle size and carbon content appeared to be decoupled as decreased particle size was observed where a shift in profile distribution of carbon content was not. Specifically, particle size decreases were most pronounced deeper in the profile and least pronounced at intermediate depths (~50 cm), a depth where many pre-urbanization profiles would be expected to exhibit their smallest particle size because of clay translocation from surface to intermediate depth soil horizons. On the other hand, change in carbon content was most pronounced at the surface (i.e. 0 cm) and persisted up to 50 cm depth. Shifts in soil carbon content covered a range from urban soils that are depleted by 50 g C·kg^−1^ to enrichment up by 20 g C·kg^−1^ relative to the mean carbon content at the surface of all urban soils analyzed. Carbon content at maximum depth (150 cm) was not altered by urbanization.

**Figure 3.**
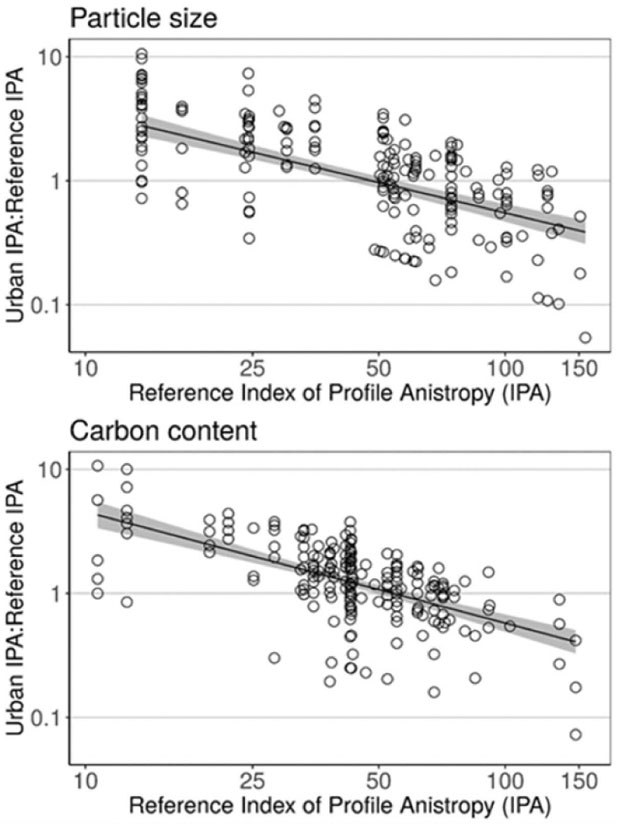
Plots show log-log linear regressions for the urban-to-reference index of profile anisotropy (IPA) values for each urban soil profile for particle size (int. = 3.15, slope = −0.81, *F*_1,204_ = 118, p < 0.001) and carbon content (int. = 3.58, slope = −0.90; *F*_1,206_ = 117). Ratios greater than 1 indicate urbanization increased profile anisotropy, equal to 1 indicate no change, and less than 1 indicate decreased profile anisotropy in urbanized soils.

Like particle size and carbon content at each depth slice, the degree of anisotropy in their entire profiles converged at intermediate levels. Specifically, urban to pre-urban IPA ratios declined from greater than 1 to less than 1 from low to high pre-urban IPA values for both particle size (log-log linear model, int. = 3.15, slope = −0.81, *F*_1,204_ = 118, p < 0.001; figure 3 (A)) and carbon content (int. = 3.58, slope = −0.90; *F*_1,206_ = 117; figure 3(B)). Ratios greater than 1 indicate urbanization increased profile anisotropy, equal to 1 indicate no change, and less than 1 indicate decreased profile anisotropy in urbanized soils. Therefore, urbanization increased IPA values in pre-urban soils with low profile anisotropy and decreased IPA values in in pre-urban soils with high profile anisotropy. Overall, results indicate that urbanization drives independent soil properties with very different pre-urban profiles towards a common degree of anistropy, creating a multi-property profile convergence. Converged levels of profile anisotropy were similar for both soil properties at IPA values of 48 and 54 for particle size and carbon content profiles, respectively. However, these values represent a decrease from pre-urban mean IPA (i.e. less complex profiles) of 65 for particle size profiles, but no change for carbon content profiles with a pre-urban mean IPA of 54. Thus, the central tendency of emergent particle size profile anisotropy was more impacted by urbanization than that of the carbon profile.

## 4. Discussion

Urbanization drives high soil disturbance in short time frames, creating new soils that have just started to form (Herrmann et al 2018). Here, we found that urbanization left an imprint that extends beyond surface soils on soil particle size and carbon content. These soil properties are of great significance to ecosystem functioning. Two major patterns were attributed to a generalized urbanization process: convergence in particle size and soil carbon content, and impacts on profile anisotropy. The imprints of urban settlements on soils discovered here show that urbanization is widely, substantially, and directly altering soils to depths greater than one meter. Thus, soil disturbances in the form of excavation, grading, and backfill linked to urbanization activities such as construction, demolition, and the installation and maintenance of subterraneous infrastructure (e.g. sewer pipes) are strong determinants of urban soil ecosystem structure.

Our findings of profile convergence facilitates two major advances in understanding the impacts of urbanization on how ecosystem-level structure is related to function. First, convergent profiles offer a prediction for how urbanization will modify soils relative to the landscape being urbanized. Urbanization acts to homogenize soils to a common profile composition. Urbanized soil ecosystem functions, as ecosystem services, are expected to follow from these structural changes. For example, in the soil profiles compared here, carbon sequestration—depending on the pre-urbanization conditions—may be modestly impacted by urbanization or dramatically increase or decrease with urbanization (suppl. Materials). Similarly, the particle size shifts shown here led to demonstrable shifts in stormwater retention capacity of the soil (Stewart et al 2019). Second, the finding of convergent profiles in important soil properties offers a straightforward and empirically-observed basis for use in process models as a standard urban end member. This is key given the past challenge of assessing and treating the often assumed heterogeneity in urban landscapes. It also means the convergent profiles are powerful as points of reference for comparative work. For example, success in ecological restoration projects could be benchmarked to these universal urban profiles for particle size and total carbon content.

Under urbanization, particle size profiles exhibit pronounced profile anisotropy arising from variation at intermediate subsurface soil depths (i.e. 30–80 cm) as a result of surface to subsurface translocation of fine textured particles. In the absence of soil formation from alluvial deposition, the soil mineral fraction is set by parent material or limited by secondary mineral formation and the vertical distribution in particle size is slowly weathered over the course of centuries to millennia (Egli et al 2018). As a result, mixing of existing soils and common introduction of imported soil fill material wholly alters any existing profile anisotropy. This all occurs without any reasonable potential to rebuild profiles that possessed functions of native soils. This is due to the long time scales involved in soil development, which are orders of magnitude longer to be useful to the human generational experience. Yet, urban landscapes can retain or rapidly rebuild soil carbon profiles like those developed under natural soil development. Organic matter amendments, agricultural management, and carbon capture from the atmosphere (Washbourne et al 2015) has the potential to greatly increase carbon in the soil surface in much shorter decadal time frames.

The patterns we found indicate urbanization shapes soils generally in the way that human development requires, which is to support built infrastructures. These objectives for stable soils involve uniformly, higher proportion of fines with non-expanding clay minerals, and low carbon content. From a geotechnical perspective, high organic matter content soils can result in instability as decomposition can result in air pockets and subsequent settling. As a result, soils used as fill material have specific characteristics, such as those set by the International Code Council. For example, soils should be evenly graded, include relatively low concentrations of carbon, and fall into texture classifications that include high amounts of silt and fine sands (International Code Council 2018, Craul 1985, ASTM International 2017).

Soil characteristics for supporting ecosystem functions and services contrast with those of these urbanized soils. Ecological management of urban settlements, thus, conflicts with not only existing urban soil profile texture and carbon content, but also with the practices that create these urbanized soil conditions. Therefore, we call for the review of established practices for constructing, deconstructing, demolishing and backfilling urban landscapes. Based on this new information, we can manage soils to maintain structural stability for redevleopment and built infrastructures. Yet, we can also look forward to creating multi-purpose urban landscapes that offer expanded suites of ecosystem services. In this way, we may navigate towards imprints of urbanization that successfully juxtapose geotechnically-and ecologically-supportive soils.

In summary, our results indicate that we can commonly represent the vertical distributions of both particle size and carbon content under urbanization, and further as a universal urban soil profile (figure 1) for these properties. Despite being blunt physical processes that shape urban soils, emergent patterns differed between soil properties. Differences in how urbanization modifies soil properties could be attributed to 1) how a property distributes in a soil profile under natural soil development and 2) how urban ecosystem management can affect the property after major earth moving activities occur. Overall, efforts to provide ecosystem services in urban landscapes must navigate the conflicting soils requirements of built versus ecological infrastructures, a topic that is arguably needed as a major interdisciplinary research frontier.

## Supplementary Material

Supplementary Material
