# Supplementary material for: Urbanization drives convergence in soil profile texture and carbon content
Source: Environ Res Lett. Author manuscript; Available in PMC 2021 Oct 14. (PMC7898117; doi:10.1088/1748-9326/abbb00)
Supplement: Supplementary Material [file NIHMS1664809-supplement-Supplementary_Material.pdf]

SUPPLEMENTAL MATERIALS

for

Herrmann, Schifman, and Shuster. Urbanization drives convergence in soil profile texture and carbon context.

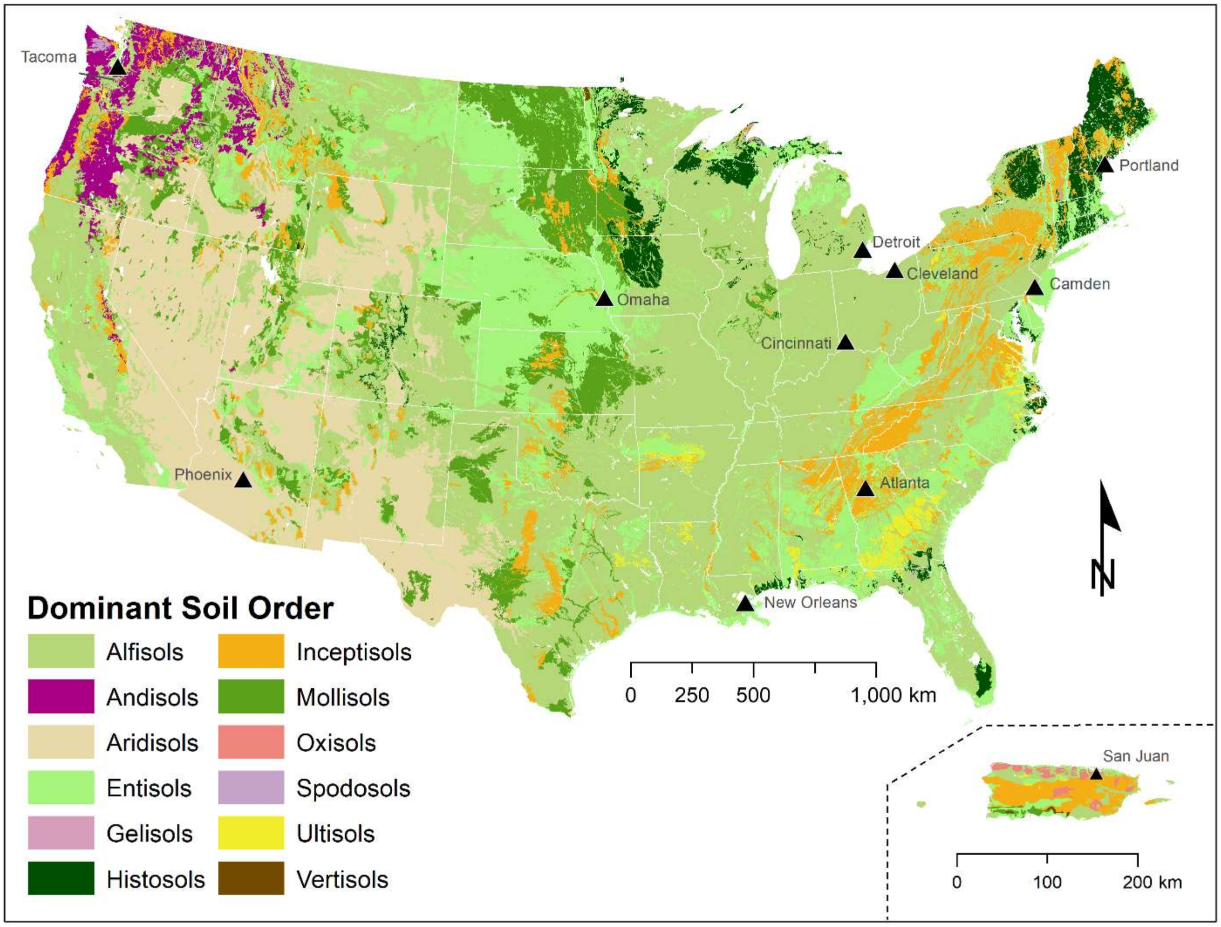

Supplemental Figure 1. Map of the contiguous continental area of the United States plus the lower right inset of the island nation of Puerto Rico show the dominant soil orders (source: U.S. Department of Agriculture, Natural Resources Conservation Service, STATSGO, 1994) and the 11 cities from which urban soil samples were taken. See Methods section 2.1 for list of cities and their soil orders.

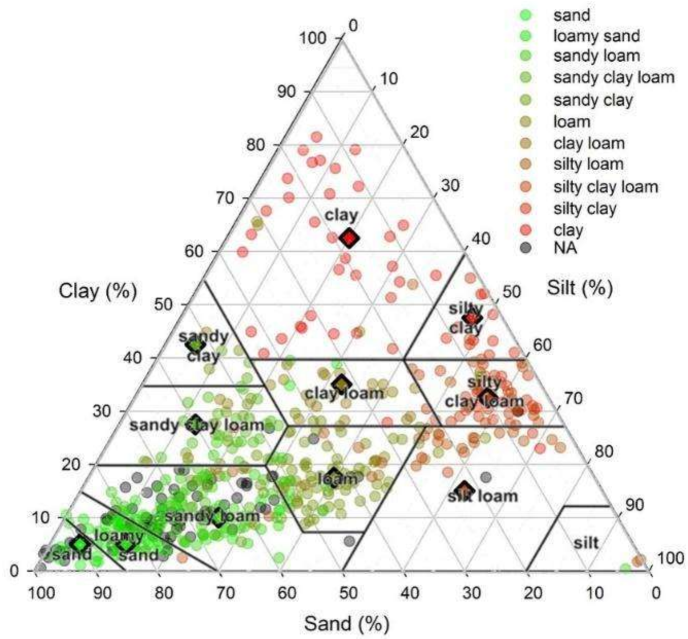

Supplemental Figure 2. Observed texture (sand, silt, clay) of urban soils (circles) and representative texture means (diamonds) used as a proxy if only texture by feel was reported for urban soils. This same designation was used for reference soils. For observed soil profiles where percent sand, silt, and clay were determined directly by the method of Gee and Or (2002), representative texture predictions were generally accurate worked best for sands, with an r^2 = 0.77, followed by clay, with an r^2 = 0.71, and silt with an r^2 = 0.58.

Supplemental Table 1. Percentages of sand, silt, and clay assigned to soil horizons based on their texture-by-feel class.

Texture class	Assigned % Sand	Assigned % Silt	Assigned % Clay
clay	17.5	20	62.5
silty clay	5	47.5	47.5
silty clay loam	10	57.5	32.5
silty loam	22.5	62.5	15
silt	7.5	85	7.5
clay loam	32.5	32.5	35
loam	42.5	40	17.5
sandy clay	52.5	5	42.5
sandy clay loam	60	12.5	27.5
sandy loam	65	25	10
loamy sand	82.5	12.5	5
sand	90	5	5

UNCERTAINTY ANALYSIS

We quantified uncertainty in modeled carbon concentrations by performing an analysis of prediction on a particularly dense data set (Detroit MI), for which carbon content was measured for all urban (n=57 soil cores, 369 soil layers) and reference (n=21 pedons, 154 soil layers) sites, and within each site, all soil horizons to 150 cm below ground surface (Supplemental Figure 3).

We applied the Root Mean Square Error (RMSE) as a metric of overall magnitude in differences between measured and estimated values:

$$RMSE = \sqrt{\frac{1}{N}\sum_{i=1}^{N}(\varphi_i' - \varphi_i)^2} \qquad (2)$$

and mean error (ME) calculations to quantify mean estimation errors as:

$$ME = \frac{1}{N}\sum_{i=1}^{N}(\varphi_i' - \varphi_i) \qquad (3)$$

Whereas in both formulas, N is the number of samples, and ϕ′, ϕ are the estimated, measured carbon values, respectively. For both error metrics, values closer to 0 indicate a better estimate; however, for the ME, a positive value indicates that modeled values are on average overestimated, and for negative values, modeled values are on average underestimated.

The predictive relationship for soil total carbon content had an RMSE of 2.66 g/kg and tended to over-estimate C based on ME = 1.98 g/kg.

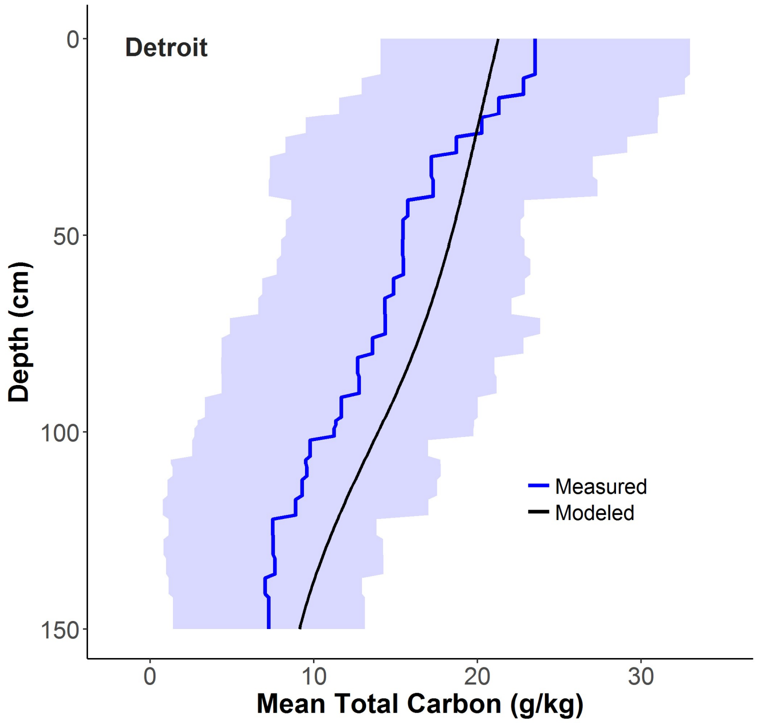

Supplemental Figure 3. Relationship between observed (measured ±95 confidence interval) and modeled soil carbon content from color value for Detroit, MI. The relationship to estimate soil carbon content from color value for urban soils was also used to obtain estimates of carbon content of reference soils.

CITY LEVEL ANALYSIS

The main article does not provide any city level comparisons. We are providing city level results and visualizations here. This is not convergence analysis as is presented in the main article. It is spline polynomial regressions with soil property profiles for particle size and carbon content group by the city and by urban vs reference classification. The dashed vertical line in the particle size (i.e., geometric mean particle diameter in mm) represents an intermediate size silt with the total size range shown on the log transformed particle diameter axis from fine clays (0.001 mm) to coarse sand particles (1 mm).

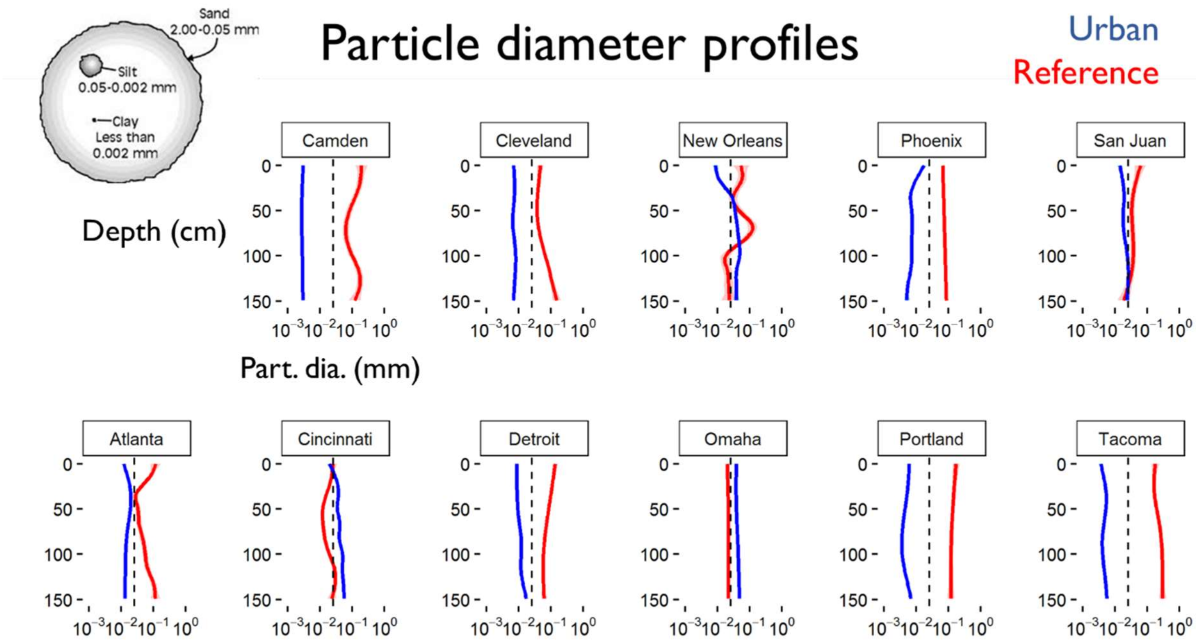

Supplemental Figure 4. Particle diameter from 0-150 cm soil depth based on regression models for urban and pre-urban reference soil profiles for each city. The dashed line is an intermediate size silt particle diameter and meant as a point of reference for comparison across graphs. The size range represented on the horizontal axes is from a fine clay on the left to a coarse sand on the right.

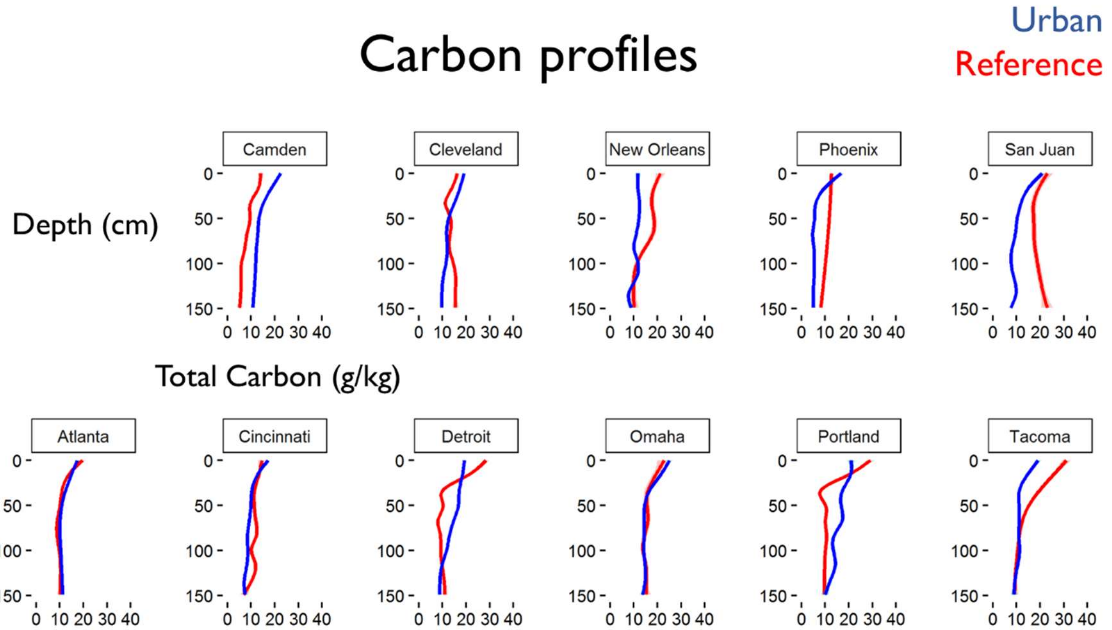

Supplemental Figure 5. Soil carbon content from 0-150 cm soil depth based on regression models for urban and pre-urban reference soil profiles for each city.

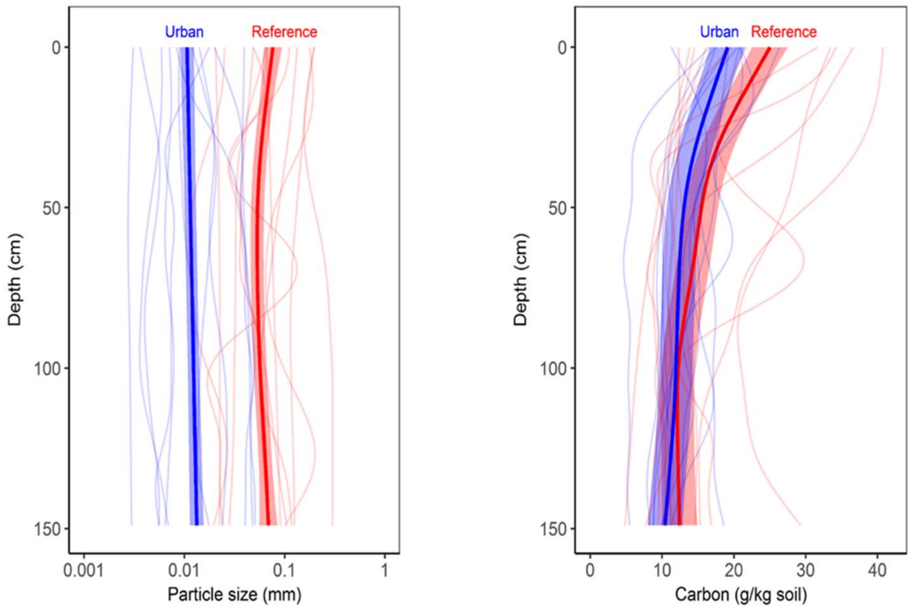

Supplemental Figure 6. Overall (i.e., global) urban versus pre-urban reference regressions based off the city-level regressions. Dark blue (Urban) and dark red (Reference) lines with shading are the modeled outputs with the 99% CI of the data shown in the light red and blue lines. Light gray lines are individual cities as shown in Supplemental Figures 4 and 5. Model parameters details are given in Supplemental Tables 2 and 3.

Supplemental Table 2. Spline polynomial regression model results for particle size profiles by city and global (i.e., model on city level modeled profiles).

Model	Parameter	Estimate	p	Effect	EDF	F	p	Model R2
Global	Intercept	-1.22	<0.001	Reference	3.08	4.44	<0.001	0.46
	Urban	-0.70	<0.001	Urban	1.00	6.55	0.01	
				City	9.17	11.14	<0.001	
Atlanta	Intercept	-1.24	<0.001	Reference	8.39	85.64	<0.001	0.75
	Urban	-0.56	<0.001	Urban	6.02	22.45	<0.001	
				Site	18.91	222.17	<0.001	
Camden	Intercept	-0.93	<0.001	Reference	7.57	51.36	<0.001	0.90
	Urban	-1.61	<0.001	Urban	3.29	4.08	<0.01	
				Site	24.85	25.00	<0.001	
Cincinnati	Intercept	-1.76	<0.001	Reference	6.15	21.71	<0.001	0.57
	Urban	0.36	0.0127	Urban	7.91	71.84	<0.001	
				Site	45.71	154.67	<0.001	
Cleveland	Intercept	-1.29	<0.001	Reference	4.61	33.58	<0.001	0.73
	Urban	-0.87	<0.001	Urban	6.52	9.95	<0.001	
				Site	78.71	283.10	<0.001	
Detroit	Intercept	-1.11	<0.001	Reference	4.45	60.21	<0.001	0.71
	Urban	-0.84	<0.001	Urban	6.91	71.81	<0.001	
				Site	69.62	181.67	<0.001	
New Orleans	Intercept	-1.40	<0.001	Reference	8.30	41.67	<0.001	0.54
	Urban	-0.10	0.591	Urban	6.87	101.94	<0.001	
				Site	22.82	130.45	<0.001	
Omaha	Intercept	-1.66	<0.001	Reference	2.00	1.77	0.124	0.67
	Urban	0.28	<0.01	Urban	4.65	32.50	<0.001	
				Site	39.85	263.56	<0.001	
Phoenix	Intercept	-1.11	<0.001	Reference	1.00	43.86	<0.001	0.93
	Urban	-1.02	<0.001	Urban	8.30	83.80	<0.001	
				Site	13.98	179.32	<0.001	
Portland	Intercept	-0.88	<0.001	Reference	3.48	20.69	<0.001	0.92
	Urban	-1.44	<0.001	Urban	6.25	55.09	<0.001	
				Site	26.96	654.89	<0.001	
San Juan	Intercept	-1.46	<0.001	Reference	4.15	12.15	<0.001	0.65
	Urban	-0.22	0.298	Urban	5.83	19.91	<0.001	
				Site	26.90	270.38	<0.001	
Tacoma	Intercept	-0.63	<0.001	Reference	5.13	23.62	<0.001	0.92
	Urban	-1.70	<0.001	Urban	6.67	20.65	<0.001	
				Site	18.95	357.04	<0.001	

Supplemental Table 3. Spline polynomial regression model results for carbon profiles by city and global (i.e., model on city level modeled profiles).

Model	Parameter	Estimate	p	Effect	EDF	F	p	Model R2
Global	Intercept	15.18	<0.001	Reference	5.34	135.23	<0.001	0.58
	Urban	-2.07	<0.001	Urban	4.35	61.75	<0.001	
				City	9.94	169.75	<0.001	
Atlanta	Intercept	10.62	<0.001	Reference	6.87	57.62	<0.001	0.58
	Urban	1.02	0.562	Urban	5.62	92.80	<0.001	
				Site	18.88	163.85	<0.001	
Camden	Intercept	8.37	<0.01	Reference	2.12	27.89	<0.001	0.48
	Urban	7.55	<0.05	Urban	7.81	70.88	<0.001	
				Site	24.74	97.13	<0.001	
Cincinnati	Intercept	77.50	<0.001	Reference	4.91	7.36	<0.001	0.45
	Urban	-1.38	0.529	Urban	7.41	100.93	<0.001	
				Site	45.54	102.62	<0.001	
Cleveland	Intercept	13.72	<0.001	Reference	4.74	3.94	<0.001	0.42
	Urban	0.09	0.961	Urban	5.79	288.16	<0.001	
				Site	77.92	76.88	<0.001	
Detroit	Intercept	13.12	<0.001	Reference	8.69	337.86	<0.001	0.54
	Urban	1.03	0.479	Urban	6.22	411.28	<0.001	
				Site	68.27	93.09	<0.001	
New Orleans	Intercept	19.48	<0.001	Reference	7.74	19.90	<0.001	0.29
	Urban	-4.71	0.126	Urban	6.35	7.60	<0.001	
				Site	22.55	48.95	<0.001	
Omaha	Intercept	25.36	<0.001	Reference	6.07	157.20	<0.001	0.57
	Urban	-7.12	<0.1	Urban	7.13	66.50	<0.001	
				Site	39.73	148.10	<0.001	
Phoenix	Intercept	10.84	<0.001	Reference	3.26	53.11	<0.001	0.73
	Urban	-4.45	<0.01	Urban	8.24	168.85	<0.001	
				Site	13.93	209.97	<0.001	
Portland	Intercept	12.24	<0.001	Reference	7.79	86.49	<0.001	0.45
	Urban	3.95	<0.05	Urban	8.28	83.85	<0.001	
				Site	26.53	61.76	<0.001	
San Juan	Intercept	25.48	<0.001	Reference	5.81	39.48	<0.001	0.61
	Urban	-13.81	<0.001	Urban	6.35	44.05	<0.001	
				Site	26.80	120.39	<0.001	
Tacoma	Intercept	16.54	<0.001	Reference	7.31	246.10	<0.001	0.68
	Urban	-5.01	<0.1	Urban	7.26	66.24	<0.001	
				Site	18.90	180.85	<0.001	
